# Naked eye direction of arrival estimation with a Fresnel lens

**DOI:** 10.1038/s41598-022-06480-5

**Published:** 2022-02-15

**Authors:** Dmytro Vovchuk, Mykola Khobzei, Dmitry Filonov, Pavel Ginzburg

**Affiliations:** 1 School of Electrical Engineering, Tel Aviv University, 69978 Tel Aviv, Israel; 2 Department of Radio Engineering and Information Security, Yuriy Fedkovych Chernivtsi National University, Chernivtsi, 58012 Ukraine; 3 Center of Photonics and 2D Materials, Moscow Institute of Physics and Technology, Dolgoprudny, Russia 141700

**Keywords:** Electrical and electronic engineering, Optomechanics, Optical physics

## Abstract

Direction of arrival (DoA) estimation is of primary importance in a broad range of wireless applications, where electromagnetic waves play a role. While a vast majority of existing techniques is based on phase lag comparison in antenna arrays, intensity-based approaches are valuable in a range of low budget applications. Here we demonstrate a direct visible to a naked eye DoA device, based on a Fresnel zone plate lens, aperture, and a light-emitting diode indicator. Being a low budget device, it still allows achieving up to 90° angle of view, 19° of angular resolution, and 11° of angular accuracy at 10 GHz operational frequency. The demonstrated approach provides fast DoA visualization and can be used to adjust point-to-point communication links, identify radio wave pollution sources at home conditions and several others.

## Introduction

The capability to detect a signal direction of arrival (DoA) is of primary importance in wireless systems, including satellite communications, radars^1^, and many others. The problem has become increasingly important with launching 5G communications, where multipath propagation is taken as an advantage^2^. A vast majority of DoA devices are based on antenna arrays, where a phase lag between the elements is monitored and used to retrieve an incidence angle and even resolve several independent sources by applying algorithmic post-processing^3^. Nowadays, sophisticated systems can deliver angular resolution well below 1° and even better than that. Those methods, however, are outside the scope of this report. Typically, phase-based detection techniques require using more sophisticated equipment in comparison with straightforward intensity measurements. Intensity-based DoA can be realized with a lens that theoretically converts an incident plane wave to a tight focus at its Fourier plane.

Microwave lensing has been intensively studied over the past 50 years^4^. A vast majority of existing configurations use either opaque metals or transparent dielectrics. While the first approach is primarily based on the interference phenomenon, the second one utilizes phase accumulation upon propagation. Dielectric lens antennas have been widely studied, and quite sophisticated shapes have been introduced^5,6^. Luneburg lens^7,8^, focusing a plane wave to a point on a spherical surface, is attractive in DoA and other applications, including satellite communications, e.g.,^9–12^. 1:1 mapping between the incident wave and the focal point on a lens’s spherical surface allows an efficient DoA detection. Additive manufacturing techniques also allow introducing quite complex shapes in new designs, e.g.,^13^. However, flat architectures have several advantages over volumetric and are used for DoA purposes. Typical designs may have either single^14^ or multi-layered layouts with metal patterns^15–19^. It is worth noting other lens-based DOA solutions, which, however, compromise between cost, performances and implementation efforts^8,20–27^ (see Supplementary material 1). Additional examples of lenses include Fresnel zone plates, as, probably, the most famous metal-based architectures^18,28,29^. Alternating transparent and opaque metal discs, capable to focus an incident wave, are among the elementary realizations. Recent works^30–32^ show a renewed interest in Fresnel zone plates and dielectric lens antennas in application to steerable antenna devices.

Our goal here is to assess a simple lens geometry and perform an intensity-based DoA that does not require using sophisticated equipment. Furthermore, we will show that a reliable detection can be performed by naked eye, i.e., by lighting a diode with RF (GHz) current.

The manuscript is organized as follows: design aspects of the device are discussed first and then followed by angular accuracy (identifying a single source) and resolution (resolving a pair) tests. Electro-optical DoA detection is demonstrated before conclusions.

## Results

### Design and numerical analysis of a single source DoA

**Figure 1:**
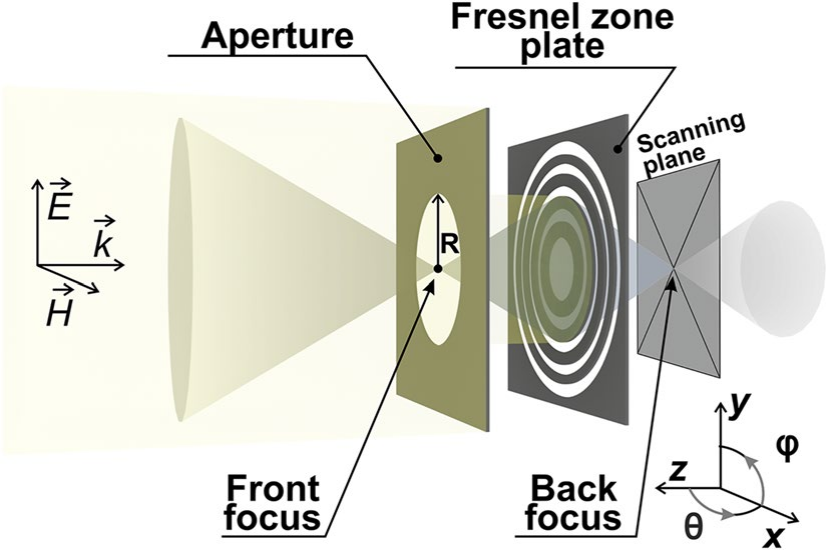
System’s layout—an aperture *in front* of the Fresnel zone plate.

The conventional Fresnel zone plate consists of a number of interchanging opaque and transparent concentric circular strips. Zone radii are given by (e.g.,^33,34^):

1 $$r_{n}^{2} = n\lambda \left( {F + \frac{n\lambda }{4}} \right),$$

where *λ* is the wavelength of the incident radiation, *F* is the lens focal distance, and $n$ is the zone’s number. The basic operation principle of the Fresnel lens is based on the interference phenomenon, which is designed to provide a focused spot on the optical axis in the case of a normal incidence. For an inclined excitation, however, the interference condition is not perfect and results in a set of aberrations, mainly including off-axial comatic and spherical aberrations^28,35,36^. Furthermore, additional parasitic interference, superimposed on the main effect, comes from edge diffraction. Chromatic aberrations are less important, as we checked numerically over ~ 20% fractional bandwidth. Conventional optical approaches to aberration minimization typically rely on cascading several convex and concave lenses^36–38^. Those systems can also include Fresnel lenses^39,40^ and further modifications with, e.g., metamaterials^41,42^. While the beforehand mentioned approaches significantly complicate a system, adding a simple aperture (order-sorting aperture in X-rays optics terminology, e.g.^43,44^) might considerably improve performances and allows suppressing higher-order diffractions. While this additional element is typically present behind the main lens, in our design it will be located in the front (Fig. 1). In DoA applications, where shallow angles of incidence are subject to detection, this input aperture allows suppressing edge diffractions and, as a result, improves the system’s accuracy and resolution, as it will be shown here. Furthermore, since the Fresnel plate is only 50% transparent, about half of the incident energy is reflected backward. Placing the aperture at the focal plane allows minimizing multiple reflections and suppressing additional undesired interference. Furthermore, the aperture performs an efficient spatial filtering at the input of the device.

After a set of optimizations (mainly on the aperture size and location), the system was designed to have the following parameters: the aperture is realized as a circular hole (*r* = 115 mm) in a thin conducting sheet (foil), nine Fresnel zones (radius of the 9th zone is *r*_*9*_ = 206 mm), calculated with the aid of (1) for an operational frequency of 10 GHz, and the distance between the lens and the aperture is 90 mm (Fig. 1). The parameters of the aperture were chosen by considering two main factors. The first one is the number of zones required for suppressing spherical and comatic aberrations. The estimation is given by^33^:

2 $$n = \sqrt {2F/\lambda } ,$$

For 90 mm focal distance, this number is approximately 2–3. The second design parameter is linked to the focus of the reflected wave. Since our goal is to map φ and θ of the incident wave (azimuthal and elevation angles that are represented at the coordinates system in Fig. 1) on the focal position in the transverse xy-plane, the radius of the aperture is directly linked with the maximal acceptance angle of the system. There is an obvious tradeoff between the system’s angular accuracy and the acceptance stereo angle for a DoA, as reducing the sorting aperture radius improves the accuracy of paraxial detection, while inclined incident waves will be primarily reflected. Relying on the above, the aperture’s radius was chosen to be equal to the size of 4th zone (*r* = *r*_4_ = 115 mm). Nine zones were taken as a performance compromise. The focal length can be further shorten to reduce the overall footprint of the system. 90 mm, however, was chosen to minimize coupling between the lens and the scanning system, as it will be discussed hereinafter.

**Figure 2:**
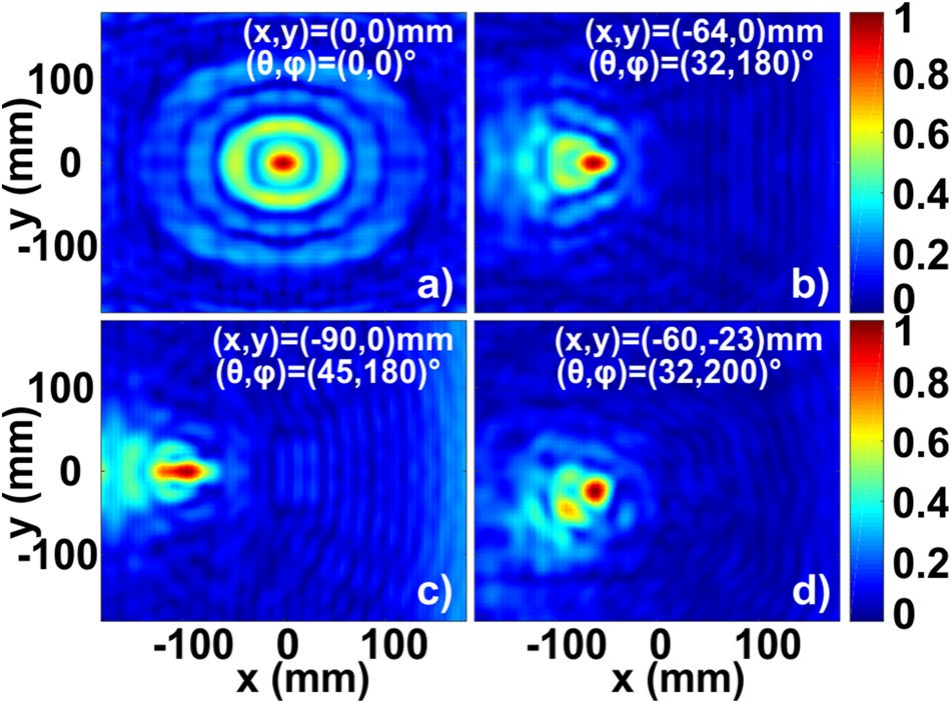
DoA of a single source—numerical results. Field intensity distribution at the focal plane (*z* = − 90 mm) is shown for four angles of incidence (in captions). Focal positions (maximal intensity) are indicated in caption.

To verify the concept, a set of numerical simulations was performed (time domain solver, implemented in CST Microwave Studio). Figure 2 summarizes the results, demonstrating the field intensity at the focal plane in the case of four different angles of incidence. A plane wave source was used to represent a signal, subject to a DoA. Intensity at the focus and the focal shift as a function of angle of incidence were maximized.

In the case of (θ, φ) = (0, 0)°, the field intensity peak is concentrated on the optical axis (x, y) = (0,0) mm. For an inclined incidence (θ, φ) = (32, 180)°, the focal spot moves to (x, y) = (− 64, 0) mm. The maximal acceptance angle (with respect to the optical axis) is approximately 90–100° (θ =  ± 45–50° in respect to z-axis). Figure 2c corresponds to (θ, φ) = (45, 180)° and (x, y) = (− 90, 0) mm. Figure 2d corresponds to (θ, φ) = (32, 200)° and (x, y) = (− 60, − 23) mm. A further increase of the elevation leads to significant interferences and makes accurate detection impossible. It can be seen that a tilted incidence leads to a focal spot distortion and intensity reduction (by a factor of up to 1.5 between maximal intensities, considering not normalized data on panels (a) and (c)). Those distortion effects are partially suppressed by introducing the sorting aperture (Fig. 1), which allows to reduce effects of tilted incidence on a bare zone plate, e.g.^45^.

After verifying that the change in an incidence angle is reflected in the focal point displacement, the next stage is to find a mapping between those two quantities. Numerical data analysis suggests 2 mm of linear peak displacement per 1° change of an incidence angle. At the same time, ray optics predicts the focus displacement along x- or y-axes as:

3 $$\theta = atan \left[ {\frac{{\sqrt {X^{2} + Y^{2} } }}{f}} \right],$$

**Figure 3:**
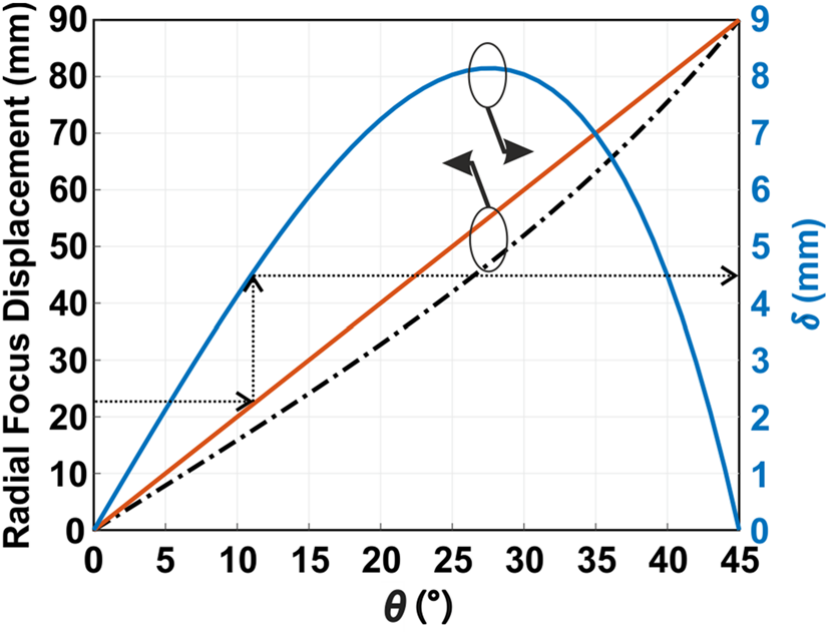
Mapping between the incident angle (θ) and the focal point displacement. The red solid line is the numerical prediction, the black dashed-dotted line is the ray optics prediction (3). Focal displacement is the left y-axis. The blue solid line is the difference (δ) between the numerical and analytical data, the corresponding y-axis is on the right side.

While this mapping is linear under the paraxial approximation, it introduces distortion for larger acceptance angles. To assess the straightforward theoretical prediction (3) with the numerical data, the focal displacement as a function of θ was evaluated—Fig. 3. The red solid and black dash-dotted lines stand for the numerical and theoretical predictions, correspondingly. The left y-axis corresponds to the radial displacement in mm. It can be seen that the data differ by a non-negligible value—the blue solid curve, the right y-axis. The maximal change is observed around 22.5°. The reason for the discrepancy is predominantly edge diffractions.

**Figure 4:**
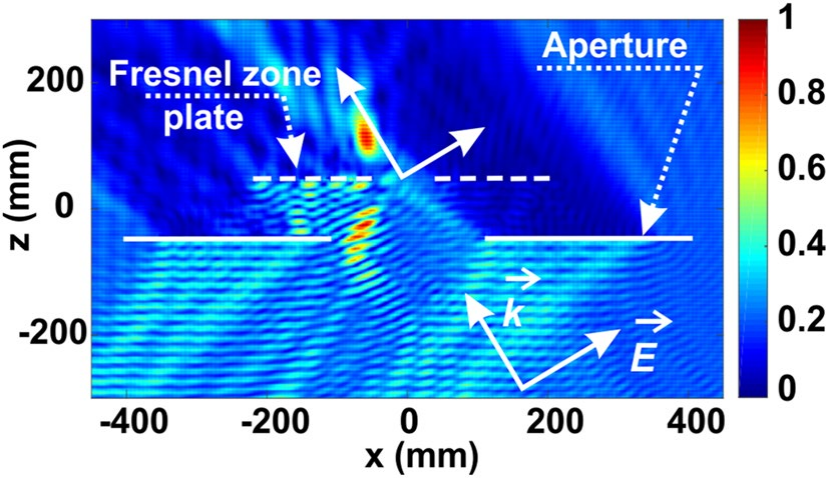
xz-plane cross-sections the normalized field amplitude for (θ, φ) = (32, 180)°.

To underline the edge diffraction effects, the cross-section of field amplitude (absolute value) at the xz-plane, corresponding to (θ, φ) = (32, 180)°, is plotted in Fig. 4. The displacement between the real focus (hot spot on the plot) and the white line (optical ray trajectory) is evident. Diffraction on the aperture is also clearly seen.

The empirical correction factor δ can be introduced into the formalism straightforwardly. Since δ-factor corresponds to a cylindrical coordinate system, different quadrants in the Cartesian system should be accurately distinguished. Finally, the set of angles is given by:

4a $$\theta = atan \left[ {\frac{{\sqrt {\left( {x - sign\left( x \right)\delta } \right)^{2} + \left( {y - sign\left( y \right)\delta } \right)^{2} } }}{F}} \right],$$

4b $$\varphi = \left[ {atan \left( {\frac{y - sign\left( y \right)\delta }{{x - sign\left( x \right)\delta }}} \right) + \pi \left[ {ceil\left( {H\left( x \right)} \right) + 1} \right]} \right]\bmod \left( {2\pi } \right),$$

where *sign(x)* is the sign of *x*, *H(x)* is the Heaviside step function, and *ceil* is the smallest integer value that is larger than or equal to a number. Using empirical results of (4), it is possible to perform a DoA quite accurately, as it will be shown hereinafter.

### Single source DoA: experimental verification

**Figure 5:**
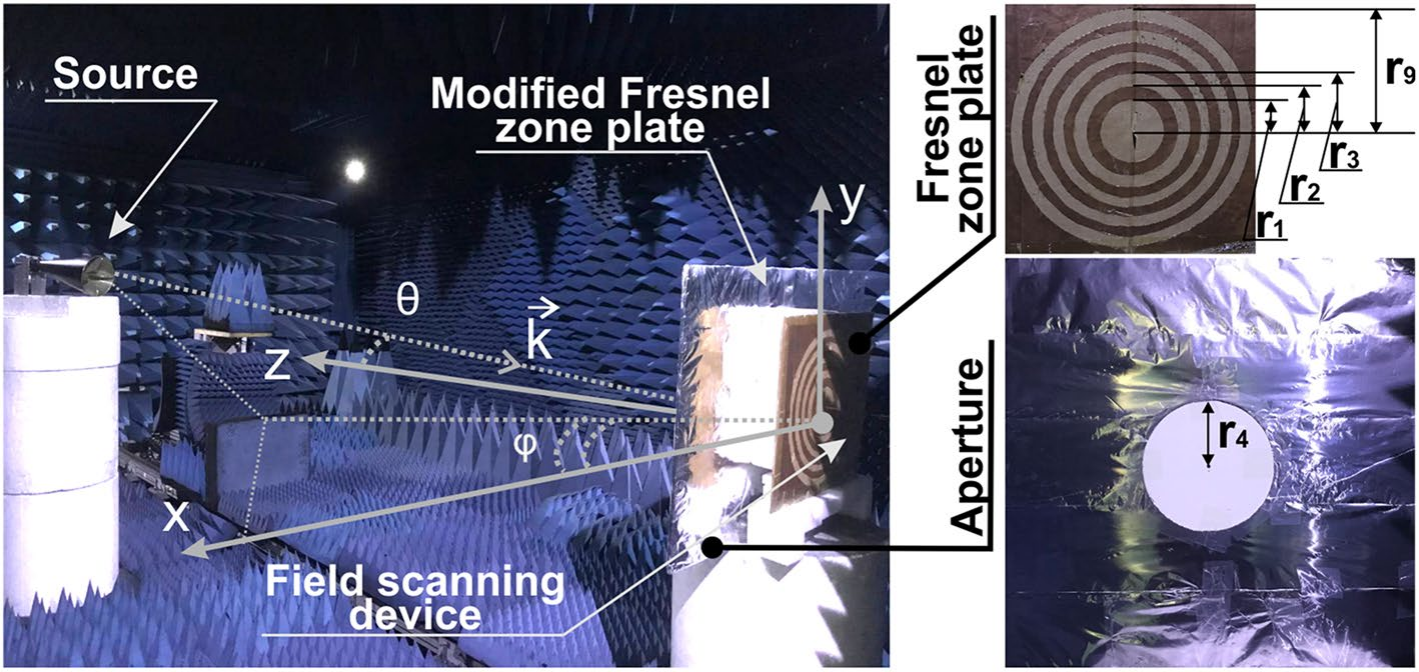
Photograph of the experimental setup—horn antenna transmits a wave that impinges the Fresnel lens-based DoA device. The loop probe scans the field at the Fourier plane of the lens. Samples are located on an RF-transparent white foam posts. Insets—photographs of the Fresnel zone plate and the aperture.

To test the beforehand-discussed design, the following experiment was performed. The setup consists of a horn antenna (source), the aperture modified Fresnel zone plate with the parameters, as they were taken in numerical simulations, and a field-scanning device (Fig. 5). The horn antenna model is NATO IDPH-2018, which covers 1–18 GHz frequency range with the corresponding gain 7.5–18 dB. The structure is set to operate at 10 GHz frequency. Opaque zones of the plate were made from a standard cooking foil (~ 0.5 mm of thickness). The foil circles were attached to a cardboard. The space between the lens and the sorting aperture was filled with RF-transparent styrofoam to ensure structure’s rigidity (Fig. 5). The transmitting horn antenna was located 2 m away from the DoA device with E-field vector polarization along x-axis and H-field—along y-axis. The radiated power is 8dBm. This setup ensures far-field operation (Fraunhofer criterion) and allows neglecting the curvature of the incident wave front, approximating the latter with a plane wave. The field at the Fourier plane of the Fresnel lens was scanned with a small loop antenna (magnetic probe antenna with 10 mm of diameter) mounted on a mechanically moving holder. The received power was around − 50 dBm, while the noise level is around − 80 dBm. Loop configuration of the probe allows making the device compact and, as a result, more precise in collecting primarily magnetic field components, polarized along the loop’s normal. It is worth noting that the lens and the aperture, having a rotational symmetry, are polarization insensitive. Consequently, either two independent measurements with the loop should be performed or a more sophisticated intensity probe can be designed. Polarization issues become important outdoors, where perpendicular to the earth polarization typically has better propagation characteristics. Performing an additional scan along the optical axis might elevate the detection accuracy, improving the localization capability of the focal spot.

**Figure 6:**
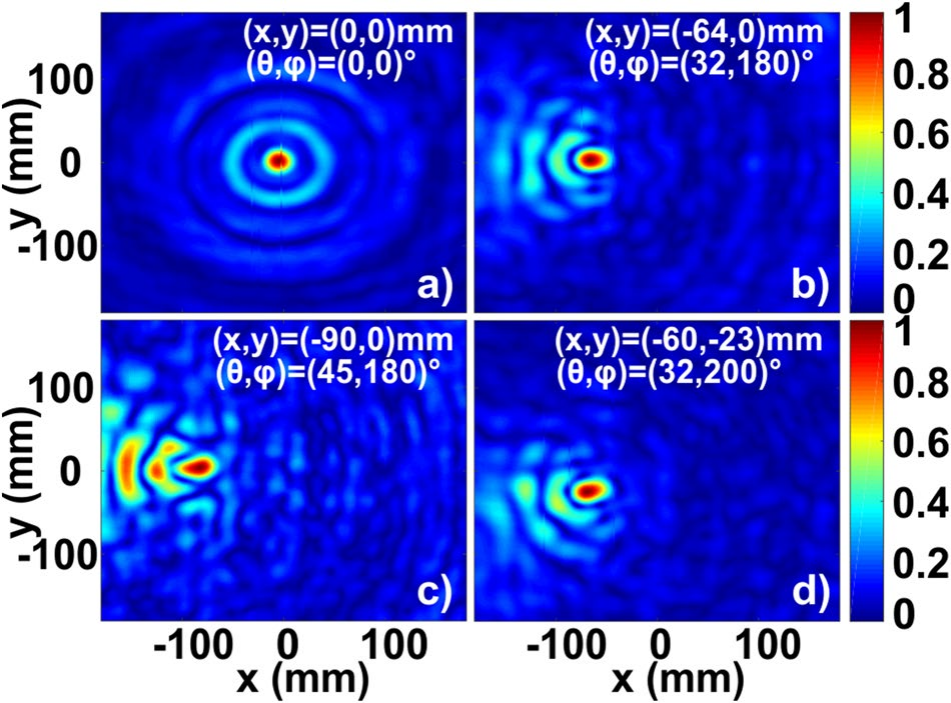
Experimental results—field intensity distributions (normalized to maximum) at the focal plane. The angle of incidence and the focal spot location are indicated in the legends.

The data acquisition was carried out with the MiDAS software, ORBIT/FR Engineering Ltd. Several source positions, characterized by angles θ and φ (refer to Fig. 5), were tested. Figure 6 summarizes the scans of the field intensity. Panel (a) for (θ, φ) = (0,0)° shows a well-defined circular focus exactly at the center of the lens and the residual interference rings. Field intensities for several other angles of incidence appear in Fig. 6, and the corresponding focal positions fit well the simulation results (Fig. 2).

To estimate the angular accuracy of the detection, the source location was swept over θ, and the Ryleigh criterion was applied. Strictly speaking, the accuracy based on peak detection depends on a signal-to-noise ratio (SNR) and can be significantly improved by enlarging the acquisition time under a white noise assumption. Here we will stick to the Ryleigh criterion without discussing high SNR techniques.

**Figure 7:**
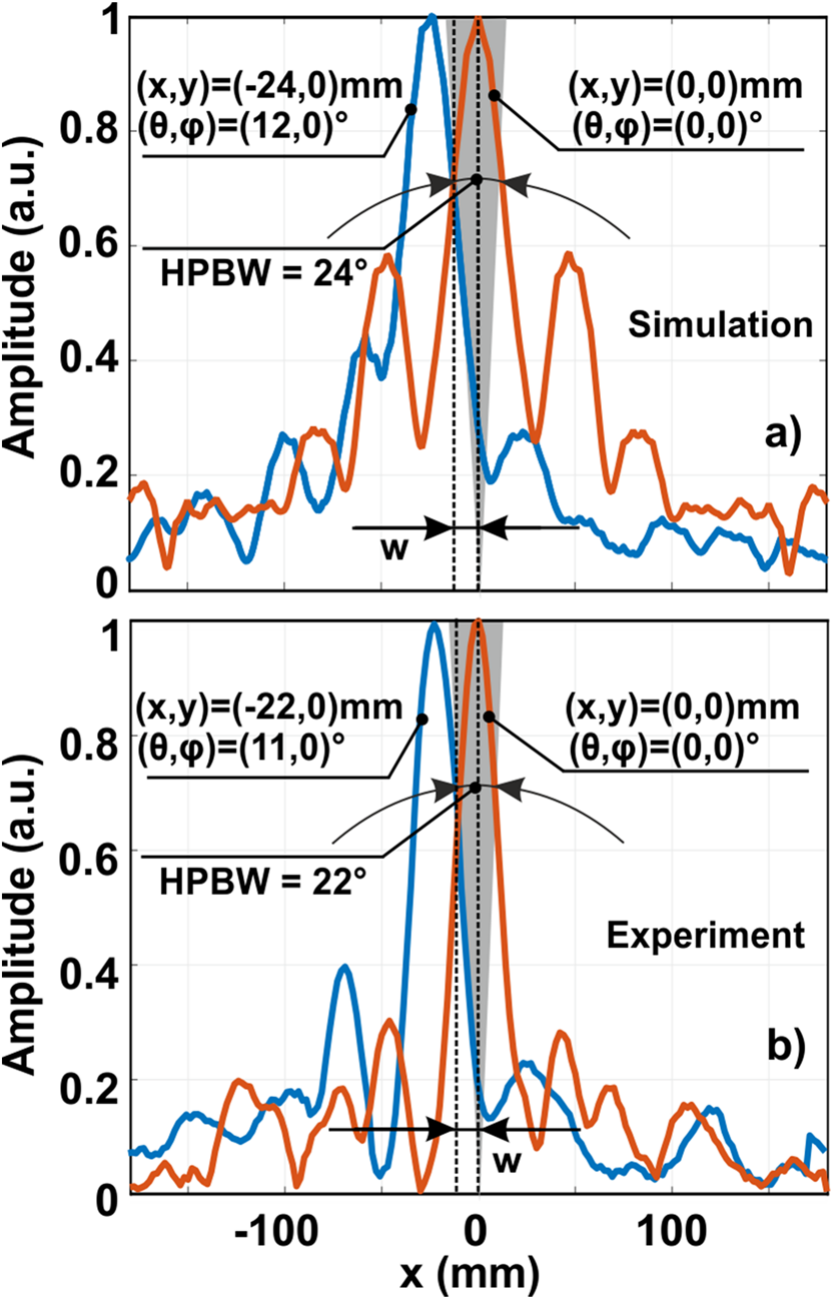
Angular accuracy—(**a**) numerical and (**b**) experimental electric field amplitude distributions for different locations of a source indicated in the plots.

Figure 7 demonstrates numerical and experimental field amplitude distribution cuts for several scenarios, i.e., a source at θ = 0°, θ = 11°, and θ = 12°. The difference between the two later cases is the shift of − 22…− 24 mm along the x-axis. The half-power amplitude analysis shows that the accuracy limit is 12° for the simulations and 11° for the experimental measurements. Similar results can be obtained by considering an analytical expression of the beam width *w* at the lens output^33^:

5 $$w = \frac{\lambda F}{{2r_{n} }},$$

where *n* = 4, therefore *r*_*4*_ = 115 mm. Thus, *w* = 11.7 mm.

### DoA: angular resolution

**Figure 8:**
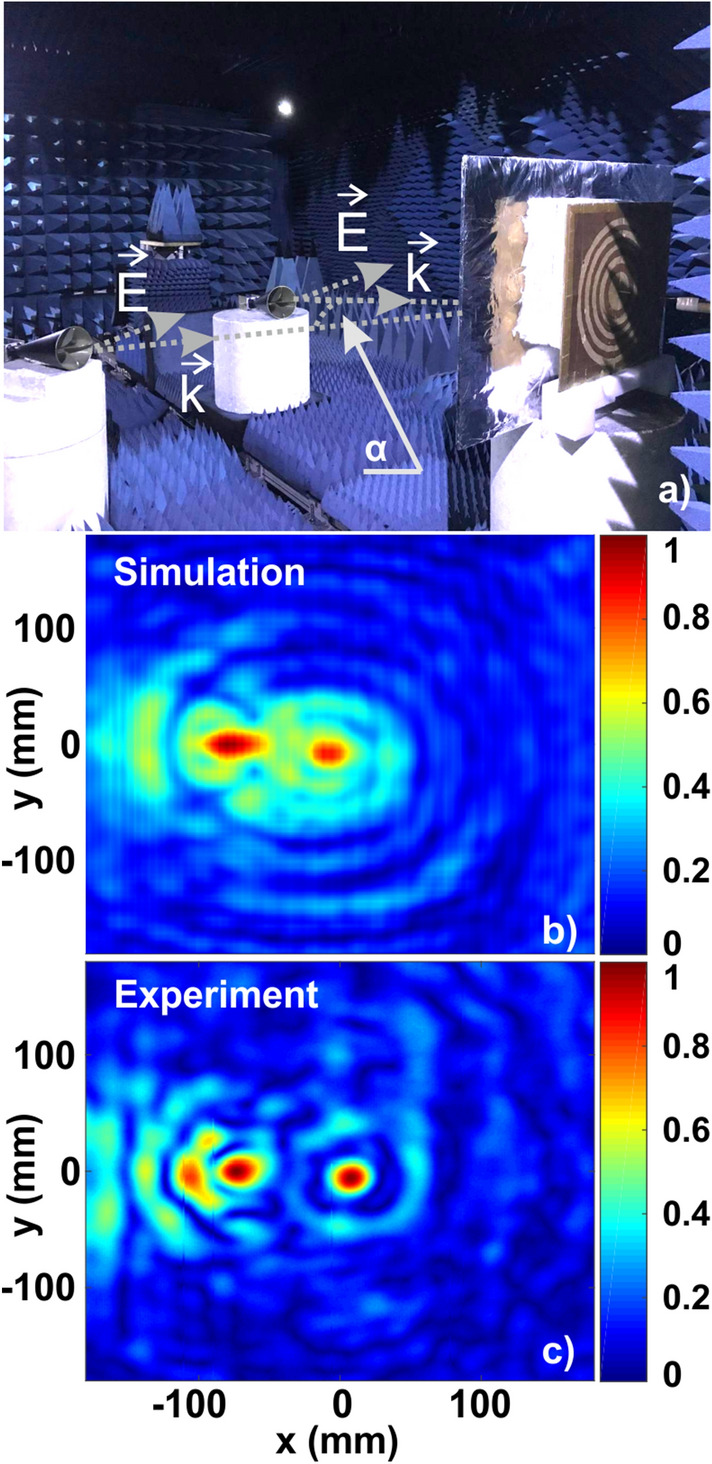
(**a**) DoA experimental setup for angular resolution. (**b**,** c**) Numerical and experimental field intensity distributions (normalized to maximum) at the lens’s focal plane.

While the previous sections concentrated on the ability to identify a single source (angular accuracy), distinguishing between several targets and even counting their number is highly important in radar applications, e.g.^1,46,47^. This capability is referred to as an angular resolution. In the case of intensity-based detection, the Rayleigh criterion can be applied in close similarity to optical imaging. The experimental setup now includes two sources—radiating antennas, placed at the far-field (Fig. 8a). Owing to the experimental constraints, both transmitters are mutually coherent and triggered by the detection system. Outdoor scenarios, however, typically rely on incoherent sources originating from different targets and undergoing different paths. In fact, mutual coherence degrades angular resolution by introducing high-intensity interference fringes. Hence, a real outdoor performance might be better than the one shown here. It is also worth noting that orthogonal polarizations can partially replicate this scenario, which was not performed here due to the realization of the polarization-sensitive detection system.

The same experimental setup, as for the angular accuracy case, was employed. The source, however, is two plane waves, radiated from identical horns. Figure 8b,c show a typical result for two sources (numerical modeling and experiment, respectively) with angular coordinates (θ, φ) = (5, 315)° and (θ, φ) = (32,180)°.

Figure 8 considers a relatively large angle (*α* = 37°) between the transmitting antennas (antennas and the lens’ normals were chosen to belong to the same plane, the electric field vectors of the two antennas are collinear) and underlines the scenario where two focal points are well distinguishable. Nevertheless, the interference fringes can be clearly seen and result from mutual coherence between the sources.

The next step is to reduce the value of *α* and apply the Rayleigh criterion. As in the case of angular accuracy, high SNR super-resolution techniques can be applied but are not discussed here^48,49^. To emphasize, the resolution is linked with Shannon’s information bound, nevertheless it depends on the linear transmission of an imaging system. In order to disentangle the super-resolution post-processing techniques from the pure performance of the system, we use the standard Rayleigh criterion for the assessment. Our detection system uses a magnetic near-field loop antenna with a 10 mm diameter aligned with the incident field polarization (H-field). Further collecting antenna size reduction can improve the resolution at the expense of SNR reduction. Angular resolution of a standard Fresnel zone plate can be calculated with^33,50^:

6 $$\beta = \Delta_{0} \frac{\lambda }{{2r_{n} }},$$

where *β* is the half-angular size of a focal spot (in radians). Δ_0_ = 1.3 is the resolution coefficient, which depends on the number of zones^33^. In our case, *λ* = 30 mm and 2*r*_*4*_ = 230 mm, leading the angular resolution to be 2*β* ≈ 19–20°.

**Figure 9:**
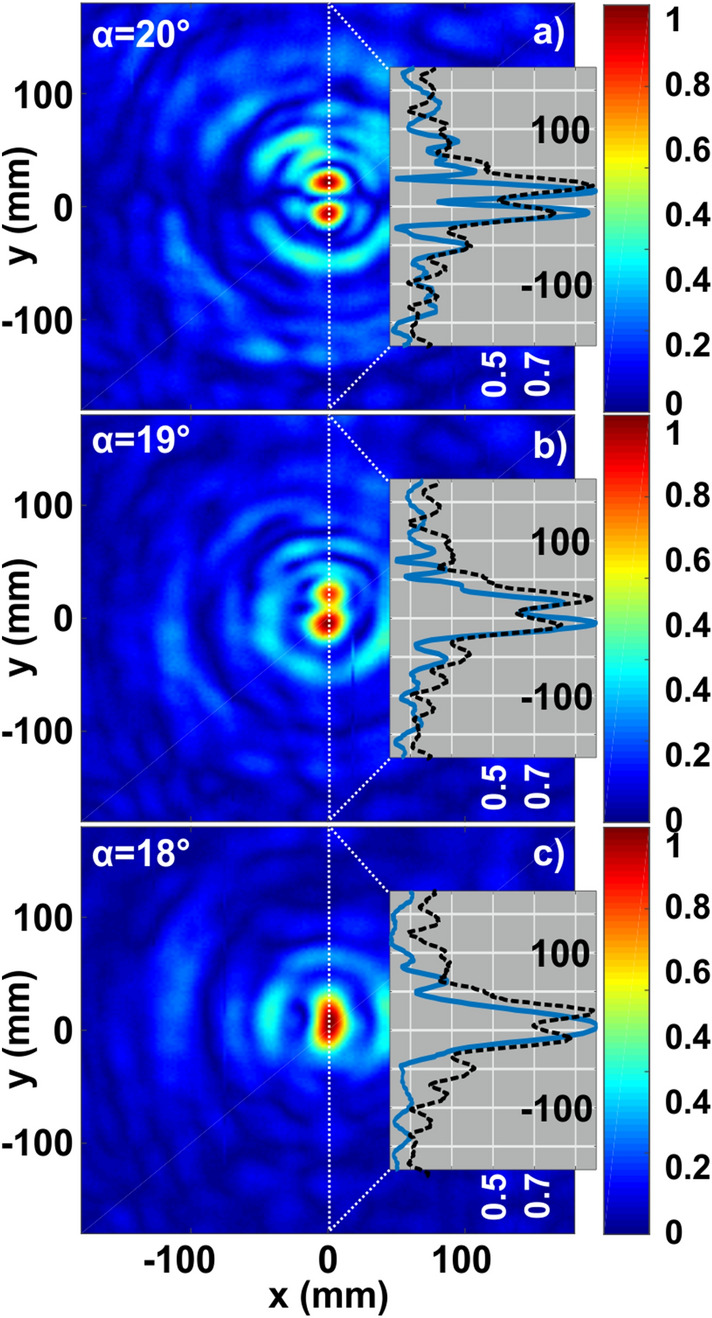
Experimental field intensity distributions (normalized to maximum) obtained at the focal plane of the lens, illuminated from two sources, separated by *α* = 20°, 19° and 18°. Insets—intensity profiles at the cut through the focal plane center (experiment—blue solid and simulations—black dashed curves).

To test the validity of this formula in application to our device, two transmitting antennas were placed closer to each other and *α* = 18–20° scenarios were assessed. Figure 9 demonstrates those three cases and explicitly underlines the resolution bounds, confirming the prediction of (6). The intensity patterns were cut at the middle and appear in the insets (solid blue curves—experiment and dashed black curves—simulations). While two peaks can be resolved for 19° and 20° sources angular separation, in 18° case only a single peak appears and comply with the Rayleigh criterion. Though, the focal spot is slightly elongated and differs from a single peak, which appears in Fig. 6a. Figure 9c shows that the theoretical and experimental limits are slightly different. While the experimental setup is not capable to resolve sources at an angle lower than 19°, theoretical bound was calculated to be 19°. It is worth noting that a digital post-processing can further increase the performance, though it cannot be done with a naked eye, as we aim here. Analog deconvolution approaches might be also applied, but require an additional apparatus.

### DoA: electro-optical detection

**Figure 10:**
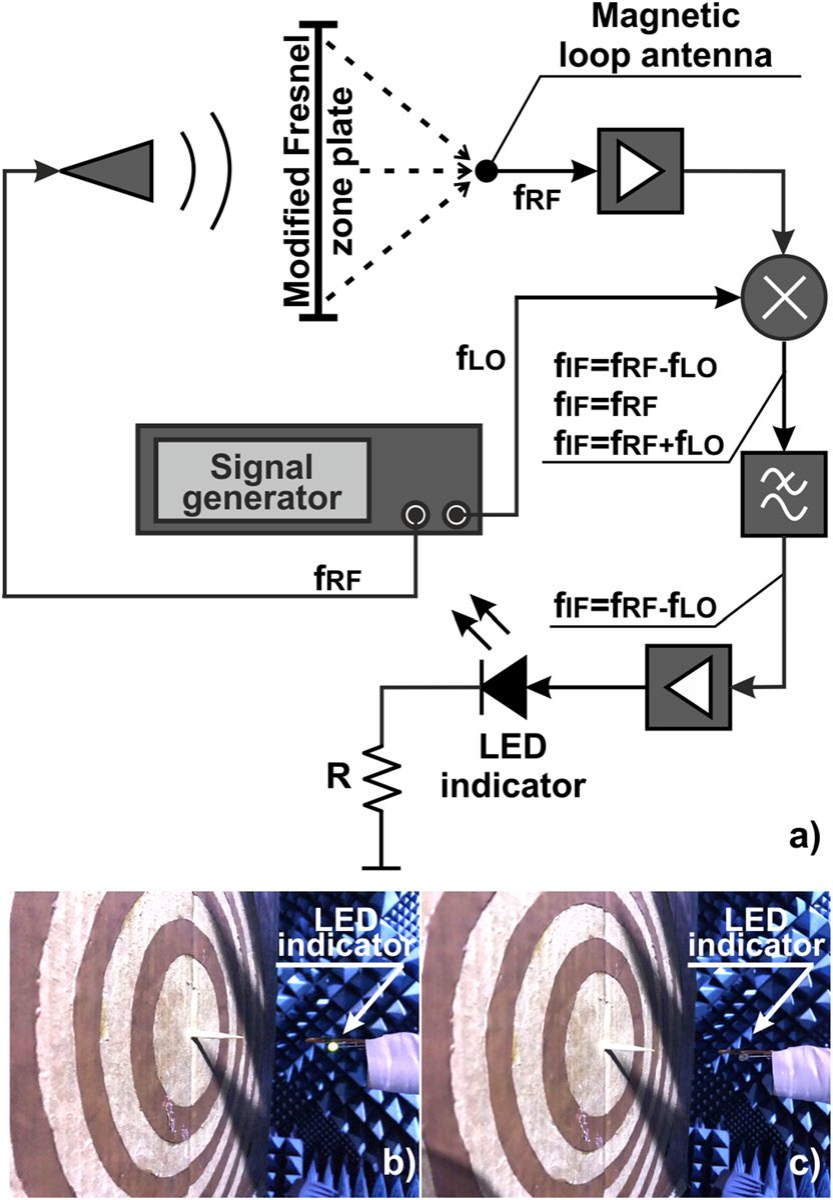
DoA—electro-optical detection. The LED indicator lights up at a focal spot, mapping the angle of incidence to the Fourier plane of the lens. (**a**) Signal amplification scheme, (**b**) light ‘on’—detection; (**c**) light ‘off’—displaced location.

While the beforehand used measurement techniques were based on a vector network analyzer, our application-oriented goal was to demonstrate a DoA device, where the detection can be made with the naked eye. Field visualization is made with a LED connected to a loop antenna. An incident field excites RF currents in the loop that is rectified and drives the LED. By predicting expected field intensities of the focal point and adjusting the resonator's quality factor, the LED will emit light only by being placed at the focus. The interference fringes will lead to weaker light emission, or even will not be sufficient to inject electrons through the diode barrier. In our case, we implemented an additional pre-amplifying circuit, which appears in Fig. 10a. The RF signal collected by the loop antenna is amplified by MWA-020180-1-4019 amplifier and then down-converted from *f*_*RF*_ = 10 GHz to *f*_*IF*_ = 100 kHz with ZX05-153-S + mixer. The low-frequency signal is then amplified again (with a custom-made low noise device) to overcome the LED’s voltage barrier of 0.7 V. An additional *R* = 200 Ω resistor is connected sequentially to control the voltage drop on the diode.

In our experimental demonstration, the LED was mounted on the scanner. A light-on point was found to indicate an accurate angular location of a source (Fig. 10b), while all the other points at the focal plane remained dark (Fig. 10c). It should be noted, however that this type of a mechanical scan is rather time-consuming. A future device is better to include multiple LEDs for a parallel acquisition. For this purpose an “RF CCD” matrix should be created, while accounting for electromagnetic cross-talks in the design (e.g. as in^26^). Another practical low-budget solution is to make a hand-scan with the single antenna. Here, a point of maximal light intensity can be found and a single source DoA can be performed. Hand-based operation, will obviously provide loser performances in comparison to a stable mechanical scan.

The overall budget of our device, based on top-grade consumables is around 100–200 USDs, with RF mixer, amplifier and filter being the main contributors. The form factor primarily defined by the lens aperture, while the overall weigh below 1 kg.

## Conclusions and discussions

A new visual approach to the DoA was suggested and demonstrated. The analysis was performed by observing intensity patterns at a Fourier plane of a zone plate lens. This configuration was functionalized with an additional filtering aperture to improve both angular resolution and the accuracy of the device. As a result, 11° and 19° accuracy and resolution were demonstrated experimentally. While this performance is far from reaching state-of-the-art solution, the overall budget of the proposed device is quite low. It makes it possible to replicate the proposal without having expensive lab equipment accessible. Furthermore, our deign can be scaled to other frequency bands, including e.g. Wi-Fi. In this case the overall size of the device will be ~ 5 times larger, while the focal position can be kept nearly the same. This type of approach might be useful in low budget applications such as crude adjustment of point-to-point communications (e.g. long range RFID^51,52^ or Wi-Fi) to identify radio wave pollution sources at home conditions. Furthermore, our approach can also find a use in heading a home internet repeater’s antenna towards a base station to improve internet connection at a countryside. The demonstrated optical DoA visualization gives an additional advantage in the fast alignment of wireless systems.

## Supplementary Information

Supplementary Information 1.

Supplementary Video 1.

Supplementary Video 2.

Supplementary Video 3.

Supplementary Legends.
