# Supplementary material for: Naked eye direction of arrival estimation with a Fresnel lens
Source: Sci Rep. 2022 Feb 15;12:2479. doi: 10.1038/s41598-022-06480-5 (PMC8847347; doi:10.1038/s41598-022-06480-5)
Supplement: Supplementary file 1 — Supplementary Information 1. [file 41598_2022_6480_MOESM1_ESM.docx]

**Supplementary material 1.**

Table 1 summarizes the comparison between several low-cost realizations of DoA, underlining several distinct differences between our design and the literature:

- *Fabrication Simplicity*. Our design comprised from cardboard and metallic foil. To compare, Luneburg lenses are manufactured with the aid of 3D-printing or chemical etching of PCBs with a high precision.
- *User operation Simplicity*. Our DoA can be performed with a hand moving indicator. It does not require any post signal processing.
- *Shape*. Our design is nearly flat (+ aperture), compared with other volumetric solutions.
- *Angle of view*. While Luneburg lens can optionally cover the entire 4pi space, practical realizations typically restrict acceptance angles, making them comparable to those, we can observe with a large aperture lens.
- *Resolution and Accuracy*. Our realization has an acceptable accuracy and resolution in cases when sources are located at the nearby.

Hereinafter, we provide a table, summarizing the technical specs.

Table 1. The comparison of the technical aspects of the existing low-cost DoA against the suggested in the paper.

| Ref. | Implementation | Operation | Geometry | Acceptance angle  Azimuth/elevation | Resolution/Accuracy | Theory/Experiment |
| --- | --- | --- | --- | --- | --- | --- |
| Our work | Cheap, materials: cardboard, foil, RF-transparent foam.  Hand-made design | Direct visible observation with a naked eye | Two thin layers (40x40cm at 10 GHz) with distance 90 mm between them | 360/90 degrees | 19/11 degrees | Theory and experiment |
| ^20^ | Low-cost, PCB chemical etching, DAQ  Board with 16 analog-to-digital channels. | signal post processing | PCB Rotman lens array + PCB dipoles array | NO/120 degrees | One object detection /~1 deg | Experiment |
| ^22^ | Dynamic metasurface antenna including phase-shifters (expensive) | signal post processing (MUSIC, beamforming) | PCB antennas array (aperture size is 18 cm at 10 GHz) | 180/- | 0.1674 rad = 9.6 deg/- | Theoretical work |
| ^8^ | Printed dielectric metasphere | signal post processing | 3D metamaterial sphere (125 mm diameter at 10 GHz) | Not declared  Our Estimate: 360/ less than 180 degrees | -/13 deg | Experiment |
| ^25^ | The Luneburg lens is a spherical structure with gradually  changing the relative permittivity + sensor tunable metamaterial absorber | NO signal post processing | 3D sphere (410 mm diameter at 2-3GHz) | 20/20 degrees | One object detection/~10 deg | Experiment |
| ^25^ | Chemically etched PCB | -- | 2D metamaterial-based Luneburg lens (120 mm diameter at 10 GHz) | NO/nor written degrees | -/5 degrees | Experiment |
| ^27^ | Printer Luneburg lens | signal post processing (correction algorithm) | 3D dielectric sphere (240 mm diameter at 4-20 GHz) | 360 deg/- | 10/10 degrees |  |
