# Supplementary material for: Naked eye direction of arrival estimation with a Fresnel lens
Source: Sci Rep. 2022 Feb 15;12:2479. doi: 10.1038/s41598-022-06480-5 (PMC8847347; doi:10.1038/s41598-022-06480-5)
Supplement: Supplementary file 5 — Supplementary Legends. [file 41598_2022_6480_MOESM5_ESM.pdf]

Supplementary information for
Naked Eye Direction of Arrival Estimation with a Fresnel Lens

Dmytro Vovchuk[1,*], Mykola Khobzei[2], Dmitry Filonov[3], and Pavel Ginzburg[1,3]

[1] School of Electrical Engineering, Tel Aviv University, 69978 Tel Aviv, Israel
[2] Department of Radio Engineering and Information Security, Yuriy Fedkovych Chernivtsi National University, 58012, Chernivtsi, Ukraine
[3]Center of Photonics and 2D materials, Moscow Institute of Physics and Technology, Dolgoprudny 141700, Russia

Supplementary material 1.
Table 1. Technical aspects of several existing low-cost DoA devices.

Supplementary material 2.
The video of one source detection. (x, y) = (0,0) mm corresponds to (θ, φ) = (0, 0)°. The LED inductor lights up at the focal spot position. The video visualizes the Fourier plane depicted on Fig. 6a.

Supplementary material 3.
The video of two sources detection with angular separation α = 37 degrees. The sources locations are (θ, φ) = (5, 315)° and (θ, φ) = (32,180)°. The LED inductor lights up at two focal spots positions. The video visualizes the Fourier plane depicted on Fig. 8c.

Supplementary material 4.
The video of two o sources detection with α = 19 degrees. The sources locations are (θ, φ) = (0, 0)° and (θ, φ) = (19,180)°. The LED inductor lights up at two focal spots positions. The video visualizes the Fourier plane depicted on Fig. 9b.
